# Self-objectification and eating disorder pathology in an ethnically diverse sample of adult women: cross-sectional and short-term longitudinal associations

**DOI:** 10.1186/s40337-019-0273-z

**Published:** 2019-12-03

**Authors:** Lisa Smith Kilpela, Rachel Calogero, Salomé A. Wilfred, Christina L. Verzijl, Willie J. Hale, Carolyn Black Becker

**Affiliations:** 1 Department of Psychiatry, University of Texas Health Science Center San Antonio, San Antonio, TX 78229 USA; 2 Department of Psychology, Western University, 1151 Richmond St, London, ON N6A 3K7 Canada; 3 Department of Psychology, Trinity University, San Antonio, USA; 4 Department of Psychology, University of South Florida, Tampa, USA; 5 Department of Psychology, University of Texas San Antonio, 1 UTSA Circle, San Antonio, TX 78249 USA

**Keywords:** Self-objectification, Body shame, Racial and ethnic minority, Longitudinal mediation, Eating disorder pathology

## Abstract

**Background:**

Extensive support exists for objectification theory’s original aim of explaining patterns of women’s mental health risk through a sociocultural lens. One pathway in objectification theory proposes a mediational role of body shame in the relationship between self-objectification and eating disorder (ED) pathology. Robust past cross-sectional research supports this proposed pathway, but largely in non-Hispanic Caucasian, college-aged samples; this pathway has yet to be empirically demonstrated longitudinally. Given previously documented concerns regarding direct measurement of body shame, we tested two measures of body shame as mediators in both cross-sectional and longitudinal models in a diverse sample of adult women.

**Method:**

Utilizing snowball sampling via email, we recruited age and racially/ethnically diverse women predominantly within the United States. Participants completed online surveys assessing self-objectification (operationalized as body surveillance), body shame, and ED pathology at baseline, 3-months and 6-months.

**Results:**

Racial/ethnic minority (*n* = 139) and non-Hispanic Caucasian (*n* = 181) adult women completed the measures. Cross-sectional moderated mediation models indicated that racial/ethnic status did not moderate relationships, and that body shame significantly mediated the relation between body surveillance and ED pathology at each time point. The longitudinal model, analyzed using cross-lagged panel analyses, was nonsignificant, as body surveillance failed to predict future body shame when controlling for past body shame.

**Conclusions:**

Racial/ethnic status did not moderate relations at any time point. Cross-sectional findings replicated past research; the longitudinal model did not support a core mediation pathway linking self-objectification to ED pathology through body shame. Because self-objectification putatively develops earlier in life, future research also should examine these relations in younger diverse samples over a longer time period.

## Plain English summary

Objectification theory proposes that sexual objectification of women in Western societies has a negative impact on their mental health. One model within this theory proposes that eating disorder (ED) pathology develops from body shame, which results from self-objectification (the internalization of these sexualizing experiences by others toward herself). Most research on this model, however, has been conducted with primarily college-aged, non-Hispanic Caucasian women. The current study set out to investigate this model in a more diverse sample of adult women over the course of 6 months. First, we found that neither ethnic grouping nor ethnic identify impacted this model. We also found that, when examined at each point in time, body shame mediated the relationship between self-objectification and ED pathology. Over time, however, this model did not hold up because self-objectification did not predict future body shame after taking into account past body shame. More research is needed to better understand how these experiences relate to one another, how they influence ED behaviors over time, and how these experiences may or may not differ in diverse samples.

## Background

Sexual objectification of women is ubiquitous in industrialized, westernized societies. Objectification theory [1] postulates that recurrent encounters of sexual objectification in Western societies condition girls and women to incorporate the sexually-objectifying gaze of others into their own self-view. This internalization is referred to as self-objectification, and is posited to be the first psychological consequence of sexual objectification for women. When women self-objectify, they tend to prioritize bodily appearance over the body’s capabilities and functionality; they also engage in chronic self-surveillance, a process that has been conceptualized as the behavioral manifestation of self-objectification [2, 3]. Objectification theory proposes that self-objectification creates more opportunities for poor subjective experiences (e.g., body shame, appearance anxiety), which may, in turn, increase women’s vulnerability to a number of mental health risks, including eating disorders (EDs).

To date, most research has tested the mental health predictions of objectification theory by focusing on ED pathology [4, 5]. Indeed, self-objectification has been found to be associated with increased body shame [6, 7] and appearance anxiety [8], as well as disordered eating [9–11]. While some research has shown direct effects of self-objectification on measures of disordered eating (e.g., [6]), the bulk of the research has demonstrated a robust indirect effect of self-objectification on disordered eating through body shame (e.g., [5, 10, 12–15]). This particular mediation pathway (i.e., indirect effect of self-objectification on disordered eating through body shame) reflects a core mediational model within objectification theory. One major limitation of existing research is that the vast majority of studies have relied on cross-sectional investigations with predominantly Caucasian samples [11]. One problem with relying on cross-sectional research to demonstrate mediation is the inability to infer causation due to the lack of temporal sequence [16]. Additionally, cross-sectional models may inaccurately detect indirect effects that are not present in longitudinal models [16]. Thus, longitudinal confirmation of mediation models is important when establishing casual and temporal mediation.

Only two studies to our knowledge have conducted longitudinal investigations of this core mediation model within objectification theory, even though the model proposes longitudinal relationships. Both studies investigated the model under conditions that could be viewed as altering self-objectification. First, Rolnik and colleagues [17] evaluated the core mediation model during a period of sorority recruitment, which was conceptualized as an objectifying experience. The model was supported cross-sectionally, but not longitudinally. Specifically, self-objectification (operationalized as self-surveillance) at Time 1 failed to predict body shame at Time 2, and Time 2 body shame failed to predict Time 3 ED pathology.

The second study [18] was conceptualized as a replication test of the first study under conditions that were viewed as being opposite to Rolnik et al. [17]. Becker and colleagues [18] tested this core mediation model during a dissonance-based body image program (see [19] for review). Results from Becker et al. [18] were very similar to Rolnik and colleagues [17]. At baseline, the model was supported cross-sectionally; longitudinally, the model was not supported as self-objectification failed to predict future body shame. It should be noted that the timing of the assessments, which lasted a little over a month in Rolnik et al. [17] and 8 weeks in Becker et al. [18], may have been too close to observe the hypothesized relationships. Furthermore, both studies investigated the mediation model in a narrow sample (i.e., predominantly White, non-Hispanic collegiate sorority members).

Consistent with Rolnik et al. [17] and Becker et al. [18], the majority of research investigating the core objectification model of ED pathology has been conducted using ethnically homogenous samples. In the limited research that counters this trend, some evidence suggests that individuals from diverse ethnic backgrounds may differentially experience various sociocultural ED risk factors, including self-objectification (e.g., [20, 21]). For instance, in a recent cross-sectional test of objectification theory across women from different ethnic backgrounds, Schaefer and colleagues [21] found cross-sectional support for this core model among Caucasian and Hispanic women, but not African American women.

As noted by Phinney [22], the construct of ethnic identity is included in one’s self-concept and is derived from the valuing of and attachment to cultural group membership. Ethnic identity – above and beyond the categorical assignment to a racial/ethnic group – may influence sociocultural risk for ED pathology in diverse ethnic groups. To further complicate this, our world is becoming increasingly multi-racial/ethnic; thus, understanding the level of ethnic identity with cultural groups is important to evaluate in the etiology of ED pathology vis a vis sociocultural risk factors. Therefore, additional research is needed to investigate the role of self-objectification and body shame in the prediction of ED pathology over time and among ethnically diverse samples.

### The current study

The present study examined a core mediation model of objectification theory linking self-objectification (measured as self-surveillance) to disordered eating through body shame both cross-sectionally at three time points (baseline, 3-month, and 6-month assessments) and longitudinally (baseline through 6-month assessment). This study was designed to address limitations in the existing literature in four distinct ways. First, in accordance with recent research on this core mediation model of objectification theory (e.g., [21]), we examined these models in a large, diverse sample of women, where just under half of the sample reported an ethnic/racial identity other than non-Hispanic Caucasian. Second, we recruited a sample that was over three times as large as previous longitudinal studies. Third, we tested the model longitudinally over a longer period of time than in previous research.

Fourth, we assessed body shame with two different measures, using both the Shame subscale of the Objectified Body Consciousness Scale (OBCS; [2]) and the Phenomenological Body Shame Scale (PBSS: [14]). The Shame subscale of the OBCS focuses on beliefs about perceived personal failures to meet internalized cultural beauty ideals around weight and shape, and directly asks about feeling ashamed, reflecting a cognitive appraisal of what women think about their appearance. This subscale is reliable and widely used as a measure of body shame in the objectification literature [3], but has two limitations.

More specifically, the Shame subscale is sometimes used in conjunction with the Surveillance subscale of the OBCS to form a composite measure of self-objectification [23], and both the Surveillance and Shame subscales include items that refer to worry and concern about one’s appearance; not surprisingly, these subscales are usually highly correlated [24]. Indeed, McKinley and Hyde [2], developers of the OBCS, proposed that self-surveillance and body shame were meant to operate hand-in-hand, as part of women’s objectified body consciousness. Thus, it is plausible that the levels of these two variables comprise objectified body consciousness collectively, but not separately. This possibility suggests that self-objectification at one time point would not necessarily predict body shame at a later time point, because the components of objectified body consciousness do not work separately over time to exert their effects, which is why researchers have found cross-sectional mediation but not longitudinal mediation. Importantly, both Rolnik et al. [17] and Becker et al. [18] used the Shame subscale of the OBCS.

In addition, according to emotion researchers, direct measurements of shame are problematic because people feel ashamed of feeling shame. One solution to this is to measure shame indirectly. For instance, the PBSS focuses on the motivational and behavioral components of experiencing body shame (e.g., a desire to hide, escape, or disappear; [25]). Although the PBSS has been used less often, it is broader in scope and also reliable [14]. By assessing a qualitatively different manifestation of body shame that shares less method variance with the Surveillance subscale than the Shame subscale, we sought to circumvent some of the concerns highlighted above with the OBCS Shame Subscale and to determine if the core mediation model would be supported longitudinally when body shame is measured differently than in previous research.

We hypothesized that race/ethnicity would moderate this core objectification theory mediation model both cross-sectionally and longitudinally. We also expected to support the core mediational model cross-sectionally at all three time points with both measures of body shame. Consistent with Becker et al. [18] and Rolnik et al. [17], we predicted that the indirect effect of self-objectification through OBCS Shame subscale would not be significant in the longitudinal model. We did, however, hypothesize a significant indirect effect for self-objectification through the PBSS longitudinally.

## Methods

### Participants

Participants were 320 women selected from a larger study of body image in a diverse racial/ethnic sample that was recruited via non-randomized snowball sampling predominantly within the United States. Participants in this subsample were selected because they completed all three time-points in the longitudinal component of the study. Participants ranged from 18 to 70 years of age (*M* = 32.46, *SD* = 11.96) and reported a mean body mass index of 25.03 (*SD* = 6.42). Participants were provided with two items, one inquiring about race and the other ethnicity, per National Institutes of Health (NIH) guidelines. In the race item, participants could select “other” or “multiple races/ethnicities.” Participants self-identified as non-Hispanic Caucasian (56.6%), Hispanic (5.0%), Asian/Asian American (11.6%), African American/Black (5.6%), multiple races/ethnicities (20.9%), or other (0.3%). Because participants could select “other” and type in a response, when participants self-selected “other” for race and typed in “Hispanic” or a country of origin currently categorized by the NIH as Hispanic (e.g., “Mexican”), we counted this response as of Hispanic ethnicity.

Twelve percent (12.5%) of participants were born outside of the United States and 5.0% currently resided outside of the U.S. Over half reported having at least a bachelor’s degree (51.4%) and 3.8% completed at least high school. Approximately one-third of participants were currently married (34.1%); 49.4% were never married.

### Procedure

Institutional Review Board (IRB) approval was granted for this study and participants were recruited via email, social media, personal and professional networks, and by word of mouth. Recruitment emails described the study as exploring body image and wellness in a diverse population of adult women, ages 18 years and older. All emails/posts requested that women forward the study invitation to their own social and professional networks. After providing informed consent, participants completed self-report questionnaires online. Participants were asked to complete the questionnaires at baseline (Time 1), 3-month (Time 2), and 6-month (Time 3) follow-up. Upon completion of the questionnaires at each time point, participants could provide their email address to enter a lottery for a $200 Amazon gift card. Although nearly 1100 women completed the baseline survey, we observed substantial attrition by 6-month follow-up (i.e., approximately 30% retention). Due to our use of longitudinal analyses and concerns with imputing approximately 60% of the data, we selected a subsample from the larger study (*N* = 320) who completed all three time points for the present study.^1^

### Measures

#### Demographic information

Participants reported their age, height, weight, race and ethnicity, country of birth, current country of residence, highest level of education, and marital status. Although self-report is not optimal for assessing height and weight, we were unable to collect these data objectively due to the online nature of the study.

#### Multigroup ethnic identity measure (MEIM)

In addition to self-reported race/ethnicity, participants completed the MEIM [22] as a measure of ethnic identity. Participants rate the extent to which they agree or disagree with 12 statements, ranging from 1 (*strongly disagree*) to 5 (*strongly agree*)*.* Higher scores indicate a stronger ethnic identity. Good reliability has been demonstrated by numerous previous studies (average α = .86; [26]), as well as by the present study (α = .87).

#### Self-objectification

We used the Surveillance subscale of the Objectified Body Consciousness Scale (OBCS; [2]) to operationalize self-objectification, which is consistent with past literature (e.g., [15, 17, 18, 27]), and assesses more of the “doing” component of self-objectification [28]. Participants rated 8 items (e.g., “I often compare how I look with how other people look”) from 1 (*strongly disagree*) to 7 (*strongly agree*), with a “not applicable” option for items that do not apply. Not applicable (NA) items are considered missing values; an individual’s subscale score was considered missing if 25% or more of responses were NA or missing. We handled reverse-scored items such that higher scores indicated greater self-objectification. Construct validity has been demonstrated for this scale [3]. In the present sample, internal consistency was good (α = .86).

#### Body shame

We utilized two separate measures of body shame in the present study. First, we assessed the cognitive components of personal body shame with the widely used Shame subscale of the OBCS, which serves as a more direct measure of body shame. Participants rated 8 items (e.g., “I would be ashamed for people to know what I really weigh”) from 1 (*strongly disagree*) to 7 (*strongly agree*), with a “not applicable” option for items that do not apply. The OBCS Shame subscale is structured and scored identical to that of the Surveillance subscale (i.e., reverse-coded items scored such that higher scores indicate greater shame; 25% or more of items NA or missing qualifies as missing overall score). In the present sample, internal consistency was good for the OBCS Shame subscale (α = .88). Second, we assessed the motivational and behavioral components of experiencing shame when participants imagine looking at themselves in a mirror, such as the desire to hide, escape, or disappear, with the Phenomenological Body Shame Scale (PBSS; [14]), which serves as an indirect measure of body shame. Participants rated 18 items (e.g., desire to hide, escape, disappear) from 1 (*never*) to 4 (*always*). Scores are averaged; higher scores indicate higher body shame. In the present sample, internal consistency was good for the PBSS (α = .96).

#### Eating disorder symptoms

We assessed ED symptoms with the Eating Disorder Examination Questionnaire (EDE-Q; [29]), the self-report version of the Eating Disorder Examination [30], which measures eating behaviors and attitudes over the past 28 days. We utilized the EDE-Q global score in this study to assess overall ED symptoms. Research indicates good internal consistency (α = .92; α = .96 in the present sample) and test-retest reliability (*r* = .90; [31]).

### Analytic strategy

#### Ethnic grouping

We grouped participants based on self-reported racial/ethnic status. As noted above, 56.6% of participants reported their race/ethnicity as non-Hispanic Caucasian. Of the 43.4% of participants who endorsed any other race/ethnicity, nearly half of these participants (48.2%) endorsed “multiple races/ethnicities.” Given 1) the small numbers of participants in any other single racial/ethnic category (which precluded simply analyzing differences between groups), and 2) the relatively high percentage of participants self-reporting “multiple ethnicities,” we collapsed the total sample into two groupings: non-Hispanic Caucasian and Multi-ethnic. Of note, although the NIH distinguishes between race (e.g., White, Black, Asian) and ethnicity (i.e., exclusively Hispanic/Latinx or Non-Hispanic/Latinx), we operationally defined “ethnicity” to be consistent with the construct of ethnic identity used within the multicultural arena more broadly (e.g., [22, 32]). Thus, from here on in this paper we use the term “ethnicity” in this broader sense, which encompasses both of the NIH categories of race and ethnicity under “ethnicity.”

In order to explore if these two groups differed in terms of self-reported ethnic identity (i.e., the strength of ethnic identity), we conducted a one-way analysis of variance (ANOVA) using the MEIM. The two groups were significantly different on MEIM (*F*(1, 318) = 46.26, *p* < .001), with the Multi-ethnic group reporting significantly greater ethnic identity (*M* = 3.50, *SD* = 0.80) than the non-Hispanic Caucasian group (*M* = 2.96, *SD* = 0.61), indicating stronger identification with ethnic grouping and culture in the Multi-ethnic group than the non-Hispanic Caucasian group.

#### Model testing

**Fig. 1:**
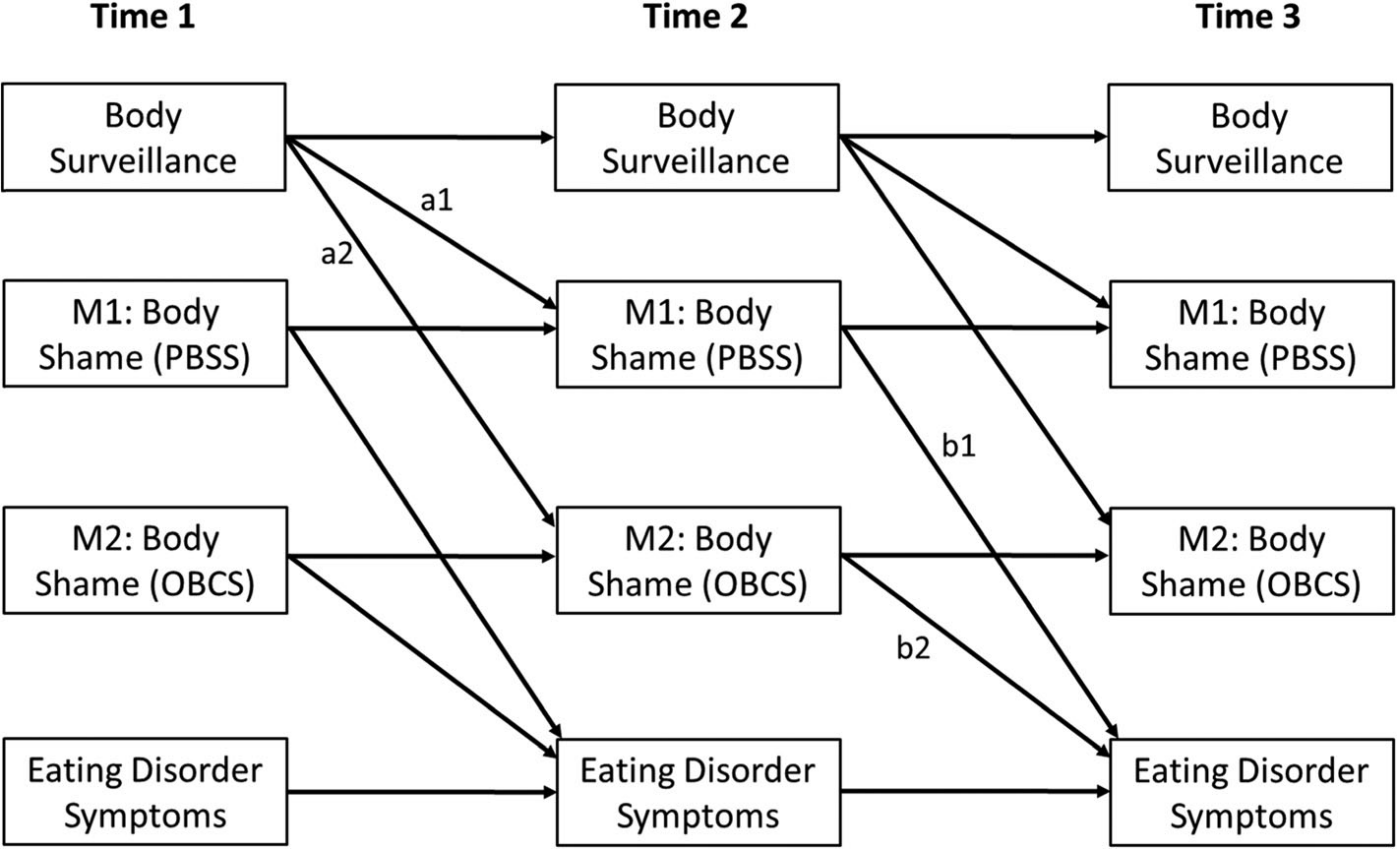
Conceptual longitudinal mediation model. Covariances within each time point were estimated but are omitted for clarity. PBSS = Phenomenological Body Shame Scale; OBCS = Objectified Body Consciousness Scale

All analyses were conducted in MPlus (Version 8; [33]). Means and standard deviations for measures at each time point are presented in Table 1. We first evaluated cross-sectional models at each of the three time points (baseline, 3-month follow-up, and 6-month follow-up) using both body shame measures as parallel mediators in order to provide the most stringent test of the indirect effects of each body shame measure. In each of these three cross-sectional models, we included body surveillance as the independent variable and ED symptoms as the dependent variable. We also tested multiple group models at each time point using ethnic group (i.e., non-Hispanic Caucasian or Multi-ethnic) as a potential moderator. Longitudinal mediation using data from all three time points was tested using the cross-lagged panel model displayed in Fig. 1, which included both body shame measures as simultaneous mediators. In the figure, the indirect effect of body surveillance on ED symptoms through body shame as measured by the PBSS is the product of the paths labeled “a_1_” and “b_1_.” Likewise, the indirect effect of body surveillance on ED symptoms via body shame as measured by the OBCS Shame subscale is the product of the paths labeled “a_2_” and “b_2_.”

**Table 1:** Descriptive statistics for sub-groups and total sample at each time point

Caucasian Sample (*n* = 181)			Multi-Ethnic Sample (*n* = 139)			Total Sample (*N* = 320)		
	*M*	*SD*		*M*	*SD*		*M*	*SD*
OBCS Surveillance			OBCS Surveillance			OBCS Surveillance		
Time 1	4.73	1.10	Time 1	4.49	1.10	Time 1	4.63	1.11
Time 2	4.59	1.09	Time 2	4.45	1.03	Time 2	4.53	1.07
Time 3	4.48	1.15	Time 3	4.38	1.09	Time 3	4.43	1.12
OBCS Shame			OBCS Shame			OBCS Shame		
Time 1	3.74	1.41	Time 1	3.44	1.26	Time 1	3.61	1.35
Time 2	3.60	1.40	Time 2	3.40	1.13	Time 2	3.51	1.29
Time 3	3.48	1.42	Time 3	3.36	1.25	Time 3	3.43	1.35
PBSS			PBSS			PBSS		
Time 1	1.83	0.70	Time 1	1.71	0.66	Time 1	1.78	0.69
Time 2	1.81	0.72	Time 2	1.69	0.64	Time 2	1.76	0.69
Time 3	1.74	0.70	Time 3	1.67	0.63	Time 3	1.71	0.67
EDE-Q Global			EDE-Q Global			EDE-Q Global		
Time 1	2.06	1.50	Time 1	1.88	1.35	Time 1	1.98	1.44
Time 2	1.88	1.44	Time 2	1.71	1.30	Time 2	1.81	1.38
Time 3	1.71	1.44	Time 3	1.66	1.30	Time 3	1.69	1.38

*Note.* OBCS = Objectified Body Consciousness Scale; PBSS = Phenomenological Body Shame Scale; EDE-Q = Eating Disorder Examination – Questionnaire

## Results

### Cross-sectional models

Ethnic group was not a significant moderator in any of the cross-sectional models. Allowing all the paths to vary across the two ethnic groups did not result in a significant improvement in model fit at baseline (χ^2^ difference (11) = 4.74, *p* = .94), 3 months (χ^2^ difference (11) = 6.88, *p* = .81), or 6 months (χ^2^ difference (11) = 4.74, *p* = .94), indicating that the groups were not significantly different on any of the paths in the model at any timepoint. Additionally, due to the aforementioned importance of considering ethnic identity, we also ran each cross-sectional model with ethnic identity (MEIM score) as a moderator on an exploratory basis; MEIM was not a significant moderator in any of the cross-sectional models (all *p*’s greater than .57). Therefore, we report the results for the full sample for each cross-sectional model (Table 2) and include confidence intervals for the indirect effects and the percent of the total effect that was mediated below.

**Table 2:** Results from cross sectional mediation models at each assessment

Time Point	Effect	b	S.E.	t
Time 1	Total Effect	0.81	0.06	14.25
	A-path (PBSS)	0.33	0.03	10.42
	A-path (OBCS)	0.79	0.05	17.24
	B-path (PBSS)	1.06	0.09	11.62
	B-path (OBCS)	0.36	0.05	7.44
	Direct Effect	0.17	0.04	4.22
	Indirect Effect (PBSS)	0.35	0.04	8.38
	Indirect Effect (OBCS)	0.29	0.04	6.78
Time 2	Total Effect	0.78	0.06	13.40
	A-path (PBSS)	0.36	0.03	11.84
	A-path (OBCS)	0.73	0.05	15.17
	B-path (PBSS)	1.02	0.11	9.48
	B-path (OBCS)	0.32	0.05	6.14
	Direct Effect	0.18	0.05	3.75
	Indirect Effect (PBSS)	0.36	0.04	8.41
	Indirect Effect (OBCS)	0.23	0.04	5.51
Time 3	Total Effect	0.79	0.05	15.11
	A-path (PBSS)	0.34	0.03	11.76
	A-path (OBCS)	0.77	0.05	15.81
	B-path (PBSS)	1.11	0.12	9.03
	B-path (OBCS)	0.28	0.05	5.30
	Direct Effect	0.20	0.04	4.68
	Indirect Effect (PBSS)	0.38	0.05	7.49
	Indirect Effect (OBCS)	0.22	0.04	4.86

*Note.* All t-values listed in the table are significant at the *p* < .001 level; PBSS = Phenomenological Body Shame Scale; OBCS = Objectified Body Consciousness Scale

At baseline, the indirect effect between body surveillance and ED symptoms was significant for both the PBSS (95% CI [.27, .43], 43.21% of the total effect mediated) and the OBCS body shame subscale (95% CI [.20, .37], 35.56% mediated). This model predicted 73.70% of the variability in ED symptoms, 28.30% of the variability in the PBSS, and 42.20% of the variability in the OBCS body shame subscale. Similarly, at 3-month follow-up (Time 2) the indirect effect was significant for both the PBSS (95% CI [.28, .45], 46.65% mediated) and the OBCS body shame subscale (95% CI [.15, .32], 29.82% mediated). This model predicted 68.00% of the variability in ED symptoms, 30.40% of the variability in the PBSS, and 36.40% of the variability in the OBCS body shame subscale. Lastly, the indirect effect was significant for both the PBSS (95% CI [.28, .48], 47.85% mediated) and the OBCS body shame subscale (95% CI [.13, .31], 27.27% mediated) at 6-month follow-up. This model predicted 73.50% of the variability in ED symptoms, 33.10% of the variability in the PBSS, and 41.10% of the variability in the OBCS body shame subscale. In summary, objectification theory was supported at all three time points using cross-sectional data.

### Longitudinal model

**Fig. 2:**
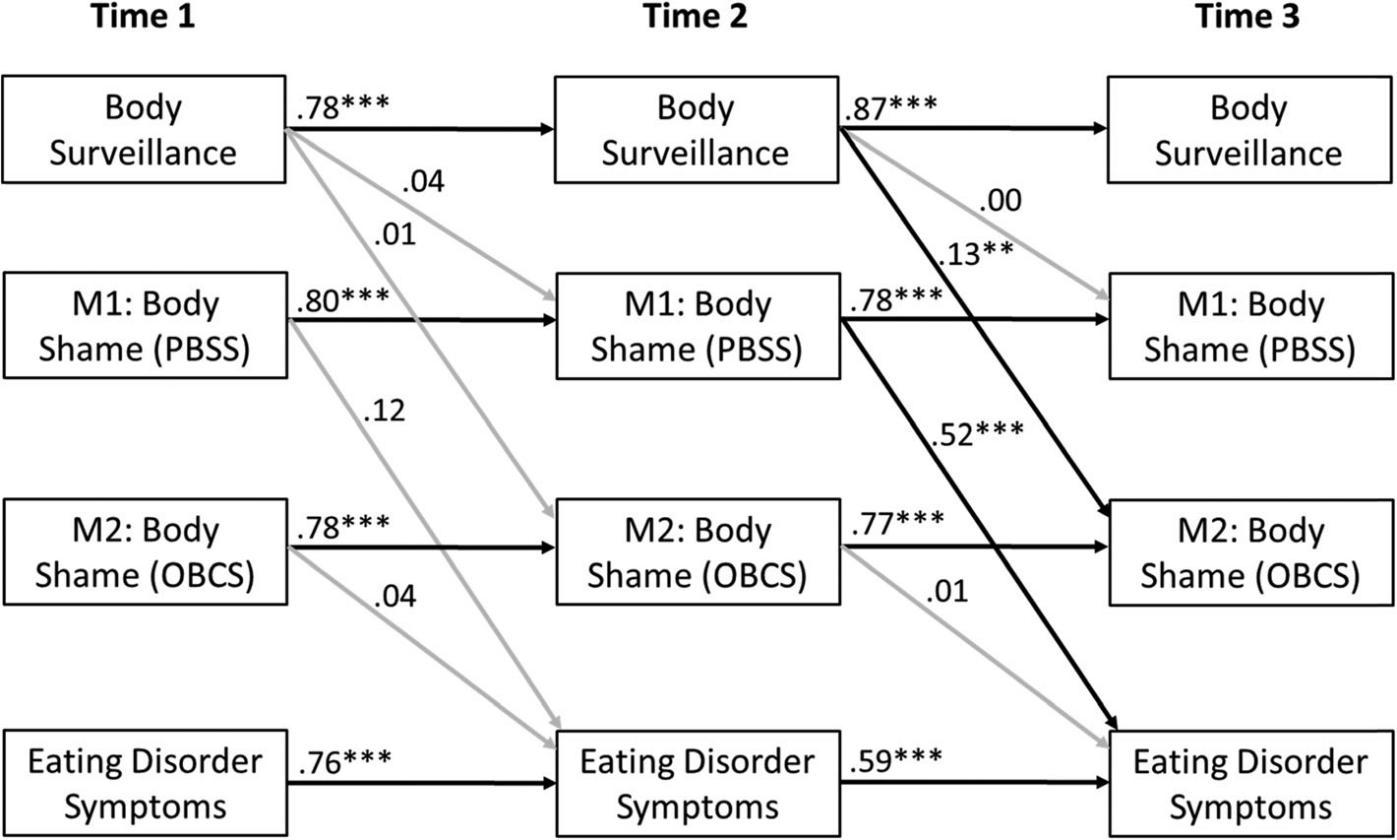
Results from longitudinal cross-lagged mediation model. Within time point covariances omitted for clarity, all were significant at *p* ≤ .005 level. Grey paths were not significant. ** *p* < .01, *** *p* < .001; PBSS = Phenomenological Body Shame Scale; OBCS = Objectified Body Consciousness Scale

Similar to the cross-sectional models, ethnic group was nonsignificant as a moderator in the longitudinal model. Therefore, we present results for the full sample. As shown in Fig. 2, all autoregressive time effects were significant. Specifically, body surveillance at Time 1 predicted body surveillance at Time 2, and the Time 2 body surveillance value predicted the Time 3 value (all *p*’s < .001). Body shame as measured by the PBSS at Time 1 predicted the Time 2 value, and Time 2 PBSS predicted the Time 3 value (all *p*’s < .001). Body shame as measured by the OBCS at Time 1 predicted the Time 2 value, as did the Time 2 value on Time 3 (all *p*’s < .001). Similarly, ED symptoms at Time 1 predicted the Time 2 value, and Time 2 ED symptoms predicted the Time 3 value (all *p*’s < .001).

Results from the longitudinal cross-lagged mediation model are shown in Fig. 2. The longitudinal mediation model included two hypothesized *a* paths (a_1_: Time 1 body surveillance to Time 2 PBSS; a_2_: Time 1 body surveillance to Time 2 OBCS body shame), neither of which was significant. Thus, the antecedent position of body surveillance in this model was not supported. Of the two hypothesized *b* paths in this model (b_1_: Time 2 PBSS to Time 3 ED symptoms; b_2_: Time 2 OBCS body shame to Time 3 ED symptoms), only the b_1_ path was significant indicating that Time 2 PBSS significantly predicted ED symptoms at Time 3. Finally, the hypothesized indirect effects between body surveillance and ED symptoms for both mediators in the model were nonsignificant.

## Discussion

The present study sought to extend the limited longitudinal research on a core mediation model linking self-objectification to eating disorder (ED) pathology through body shame by testing the model under the following conditions: 1) evaluating the core mediational pathways in a diverse, multi-ethnic sample of adult women across the lifespan, 2) using a larger sample than in previous research, 3) examining two measures of body shame that tap into different aspects of body shame in the context of objectification theory, and 4) testing the theory over a 6-month time period. Regarding ethnicity findings, results suggest that the relations between the variables detailed in the core mediation model operated similarly in Multi-ethnic, predominantly American participants as compared to non-Hispanic Caucasian participants. This finding is somewhat inconsistent with recent research by Schaefer and colleagues [21], who found that body shame did not mediate the relationship between self-objectification and eating disorder pathology in African American women. However, the inconsistency may be an artifact of differing methodology. Schaefer et al. [21] examined three distinct racial/ethnic groups, non-Hispanic Caucasian, African American, and Hispanic, whereas this study grouped different non-Caucasian races/ethnicities together. We did so primarily because many participants did not identify with a single race/ethnicity and therefore did not have adequate power to examine individual differences between specific ethnic and racial groups. Thus, the present study may have masked differences between single ethnic groups.

Consistent with both our hypotheses and past research (e.g., [21]), results from the cross-sectional mediation models at each time point (i.e., baseline, 3 months, and 6 months) indicated that both measures of body shame mediated the relation between self-surveillance and eating disorder (ED) pathology, thus providing robust support for this core mediation model within the broader objectification theory. Of note, much of the past research examining these relationships has been conducted in predominantly non-Hispanic Caucasian samples, and frequently utilized college-aged women as participants. In the current study, however, our sample was comprised of nearly 50% multi-racial/ethnic women with ages spanning much of the lifespan (i.e., 18–70). Therefore, our results with a more diverse sample of women further support prior cross-sectional relations between self-surveillance, body shame, and ED pathology.

With regards to our longitudinal hypotheses, results were mixed. Consistent with past research (e.g., [17, 18]) and our hypothesis, body shame operationalized via OBCS Shame did not mediate the relation between self-surveillance at baseline and ED symptoms over time, when controlling for prior levels of both body shame and ED symptoms. Specifically, a cross-lagged panel analysis indicated that self-surveillance at baseline did not significantly predict body shame using the OBCS Shame subscale at 3 months when controlling for baseline body shame. OBCS Shame at 3 months also did not predict ED pathology at 6 months.

Contrary to our hypotheses, the longitudinal model also was not supported when using the PBSS. Specifically, baseline self-surveillance again did not significantly predict body shame at 3 months. Of note, although self-surveillance did not predict later PBSS, the PBSS was a better predictor of ED pathology at 6 months than the OBCS Shame subscale (Fig. 2). Therefore, the PBSS may tap into an aspect of the body shame construct that is more predictive of ED behaviors over time. The PBSS also may have better construct validity for measuring body shame as described in objectification theory than does the OBCS Shame subscale [14].

One interpretation of the longitudinal findings is that objectification theory is simply incorrect with regards to the proposed relationship between self-objectification, body shame and ED pathology. We argue, however, that this is a problematic conclusion to draw at this time for several reasons. First, it is premature to abandon this pathway of objectification theory given the very limited body of longitudinal objectification research. Second, objectification theory posits numerous pathways linking sexual and self-objectification to other intermediary variables in the prediction of disordered eating. We did not test the full objectification theory model in the present study, and therefore we cannot draw any conclusions about the veracity of objectification theory to predict ED pathology based on extant findings. Finally, a number of factors could account for the fact that we did not find longitudinal support for the indirect effect of self-surveillance on ED pathology through body shame, and these factors should be addressed in future research.

For instance, it may be that the transaction of self-surveillance and body shame occurs at a substantially faster rate, and also may co-occur, which is consistent with our cross-sectional findings. As such, other methodology (e.g., ecological momentary assessment, or EMA) may be better at detecting longitudinal relationships. For example, Fitzsimmons-Craft and colleagues [34] found that state-level body surveillance was a proximal trigger of body dissatisfaction among college women, when assessed multiple times per day over a two-week period.

Moreover, in adult women, the effects of and reactions to chronic self-objectification (i.e., subsequent body shame) may be firmly established prior to assessment in the study. Given that women and girls experience sexual objectification beginning in youth in Western cultures, discernable and causal longitudinal effects of self-surveillance on body shame may be conflated by the time one reaches adulthood. Thus, we may be trying to longitudinally document a process that occurred years earlier and is not ongoing. Typically, with longitudinal studies, the outcome of interest has not yet occurred at baseline. In this case, a true predictive longitudinal investigation of the development of ED pathology would need to assess self-objectification and body shame prior to the occurrence of ED pathology, and self-objectification prior to the occurrence of body shame. In the sample of community women examined in the present study, ED pathology co-occurred with self-objectification and body shame, as observed with the cross-sectional findings. As such, we recommend that researchers consider a much more comprehensive risk factor study, which assesses self-objectification in a younger sample without any evidence of ED pathology, and then follows participants over an even longer time period to observe if self-objectification predicts the development of ED pathology via body shame. As long as participants are assessed repeatedly, such a study could test both the longitudinal association of self-objectification and ED pathology and whether or not shame mediates that relationship.

In addition, although the OBCS Surveillance subscale is a commonly used operationalization of self-objectification, this measurement scale has been criticized on conceptual and methodological grounds as a valid indicator of self-objectification [3, 35]. In particular, the content of the scale is narrowly focused on worry and surveillance around clothing and general appearance, and does not adequately discriminate itself from body shame and other body image scales. Self-objectification involves a doubled perspective on the self whereby women come to view themselves from the outside as well as the inside, disrupting the self-body connection—none of which is captured in extant measures of self-objectification [3]. Critically, these narrowed operationalizations contribute to inconsistent and inaccurate interpretations of the evidence for the role of self-objectification across the various domains in which it has been tested. While cross-sectional findings shore up support for the role of self-objectification in models of ED pathology when the OBCS Surveillance subscale is used, the null longitudinal findings observed here and elsewhere behoove researchers to consider and select the assessment of self-objectification carefully in future studies.

Lastly, our results indicate that past body shame is a very robust predictor of future body shame, which is consistent with past literature [17, 18]. Thus, it is possible that although early life experiences of self-objectification may have played a role in the initial development of body shame, perpetual body shame is more influential in maintaining levels of body shame than is continued self-surveillance. The above proposed risk factor study could provide evidence as to whether or not this is the case.

There are numerous limitations to the current study. First, due to extremely high attrition after the baseline assessment (upwards of 60% attrition), the present study utilized completer analyses. Therefore, self-selecting out of the study may have been systematically affiliated with levels of body shame, self-surveillance, or ED behaviors. Feedback from some participants who opted out of completing follow-up assessments, however, stated that the time to complete the survey was not feasible for them. Additionally, we only used survey/self-report data that were completed online and not in a controlled environment. Also due to low numbers of participants in specific ethnic and racial groups, we were unable to examine specific cultural/ethnic differences across various groups. Within the multi-ethnic group, however, nearly 50% of participants self-described as multi-racial or “other,” therefore narrowing racial or ethnic identity of these participants into one category would not have been accurate, either. Future research would benefit from a more thorough investigation of relations between these constructs in various ethnic and racial groups.

## Conclusions

Overall, results from the current study using a more diverse sample provide further support for the cross-sectional relations of self-surveillance, body shame, and ED symptoms. These models held up at each time point, thus these relations operated similarly at each time point in the current study. Longitudinal results, however, did not support the core meditational model described by objectification theory; yet, findings are consistent with two other longitudinal evaluations of these relations. Limitations hinder broad generalization of the current findings; however, future research should examine relations of these variables over time in order to thoroughly examine the causal and/or cyclical relations in the core mediation model of disordered eating described within objectification theory.

## Data Availability

The datasets used and/or analyzed during the current study are available from the corresponding author on reasonable request.

## Abbreviations

ANOVA: Analysis of Variance
ED: Eating Disorder
EDE-Q: Eating Disorder Examination - Questionnaire
IRB: Institutional Review Board
MEIM: Multi-Ethnic Identity Measure
NIH: National Institutes of Health
OBCS: Objectified Body Consciousness Scale
PBSS: Phenomenological Body Shame Scale

## Footnotes

1 Given the use of completers in this study, we conducted attrition analyses in which we compared completers versus non-completers on baseline (Time 1) measures used in the model testing (i.e., body surveillance, both measures of body shame, and ED pathology) using one-way analyses of variance. There were no significant differences between completers and non-completers on any of these variables (all *p* values > .40).
